# Investigation of clinical and genetic characteristics of Alport syndrome using a national registry in Japan (JP-ALPS)

**DOI:** 10.1007/s10157-025-02758-w

**Published:** 2025-09-19

**Authors:** Yusuke Okuda, Naoaki Mikami, Riku Hamada, Hiroshi Hataya, Kazuki Tanaka, Chikako Terano, Naoya Fujita, Kenichiro Miura, Kiyonobu Ishizuka, Yoko Shirai, Koichi Kamei, Masao Ogura, Takayuki Okamoto, Ryota Suzuki, Shunsuke Shinozuka, Yuko Shima, Masafumi Oka, Wataru Shimabukuro, Hiroyasu Tsukaguchi, Tetsuji Inagaki, Kei Nishiyama, Taeko Hashimoto, Naoko Ito, Tomohiko Yamamura, Tomoko Horinouchi, Kenji Ishikura, Koichi Nakanishi, Kandai Nozu

**Affiliations:** 1 Department of Pediatrics, Kitasato University School of Medicine, 1-15-1 Kitazato, Minami-ku, Sagamihara, Kanagawa 252-0374 Japan; 2 Department of Nephrology and Rheumatology, Tokyo Metropolitan Children’s Medical Center, Tokyo, Japan; 3 Department of General Pediatrics, Tokyo Metropolitan Children’s Medical Center, Tokyo, Japan; 4 Department of Pediatric Nephrology, Aichi Children’s Health and Medical Center, Aichi, Japan; 5 Department of Pediatric Nephrology, Tokyo Women’s Medical University, Tokyo, Japan; 6 Division of Nephrology and Rheumatology, National Center for Child Health and Development, Tokyo, Japan; 7 Department of Pediatrics, Hokkaido University Graduate School of Medicine, Sapporo, Japan; 8 Department of Pediatrics, Matsudo City General Hospital, Matsudo, Japan; 9 Department of Pediatrics, Wakayama Medical University, Wakayama, Japan; 10 Department of Pediatrics, Faculty of Medicine, Saga University, Saga, Japan; 11 Department of Child Health and Welfare (Pediatrics), Graduate School of Medicine, University of the Ryukyus, Ginowan, Japan; 12 Second Department of Internal Medicine, Clinical Genetics Center, Kansai Medical University, Hirakata, Japan; 13 Department of Nephrology, Miyagi Children’s Hospital, Sendai, Japan; 14 Departments of Pediatrics, Graduate School of Medical Sciences, Kyushu University, Fukuoka, Japan; 15 Yume Pediatric Clinic, Kasugai, Japan; 16 Department of Surgical Pathology, Tokyo Women’s Medical University, Tokyo, Japan; 17 Department of Pediatrics, Kobe University Graduate School of Medicine, Kobe, Japan

**Keywords:** Alport syndrome, Registry, eGFR slope, Renin-angiotensin system inhibitors

## Abstract

**Background:**

Comprehensive epidemiological information regarding Alport syndrome, particularly from national cohorts, is limited.

**Methods:**

Utilizing a national Alport syndrome cohort in Japan established in October 2022, we analyzed clinical characteristics according to genotype. Only baseline data collected retrospectively at enrollment were used. We present longitudinal trends in estimated glomerular filtration rate (eGFR) and urine protein-to-creatinine ratio.

**Results:**

Of the 121 patients included, 105 (86.8%) underwent genetic testing and 82 (67.8%) had a kidney biopsy. Among those with genetic testing, 77 (73.3%) had X-linked Alport syndrome. Kidney function was normal at disease onset, with a median eGFR of 112.9 (interquartile range, 99.3–131.1) mL/min/1.73 m^2^. Although a steep decline during adolescence was observed in some male patients with X-linked Alport syndrome, eGFR decline was relatively slow during childhood and adolescence; the point estimate of eGFR at age 20 was 88.6 mL/min/1.73 m^2^. Six patients transitioned to end-stage kidney disease during the follow-up period. Eighty-one patients (66.9%) used renin-angiotensin system (RAS) inhibitors, and the rate of eGFR decline was slower after RAS inhibitor initiation. Notably, the median ages at onset and diagnosis were 3.0 and 5.1 years, respectively, because Japan’s widespread urinalysis screening program for 3-year-old children enables initiation of early treatment.

**Conclusions:**

In our cohort, which consisted mainly of patients who did not require kidney replacement therapy in childhood and adolescence, kidney function was preserved throughout this period except for some male patients with X-linked Alport syndrome. RAS inhibitor use may be associated with a reduced rate of eGFR decline.

**Supplementary Information:**

The online version contains supplementary material available at 10.1007/s10157-025-02758-w.

## Introduction

Alport syndrome is a hereditary progressive disease affecting the kidneys, often accompanied by sensorineural hearing loss and ocular abnormalities. Previous genetic advancements have revealed that Alport syndrome is caused by pathogenic variants in genes encoding type IV collagen. Although diagnosis was previously based on pathological findings and clinical information, including family history, non-invasive genetic testing has become the recommended diagnostic procedure in recent years [1]. It is classified into X-linked (XLAS), autosomal recessive (ARAS), and autosomal dominant (ADAS) forms, accounting for approximately 80%, 15%, and 5% of cases, respectively [2]. XLAS and ARAS are often present in childhood and tend to have a more progressive course, whereas ADAS generally has later onset and milder progression.

Prior studies of Alport syndrome focused on clinical characteristics associated with kidney disease, including pathological findings [3–5], hearing loss [6–8], and ocular abnormalities [9–11]. Advances in genetic research have also led to the identification of genotype–phenotype correlations [12–14]. This clinical and genetic evidence has substantially contributed to the understanding and management of Alport syndrome. However, comprehensive epidemiological information regarding Alport syndrome, particularly from national cohorts, remains limited. In Japan, a nationwide urinalysis screening system facilitates the early detection of urinary abnormalities in children, enabling earlier diagnosis and intervention in kidney diseases, including Alport syndrome. National registries in Japan can leverage this system to collect longitudinal clinical and genetic data from pediatric patients across the country.

We established a national registry database for Alport syndrome in Japan. This study was performed to provide comprehensive epidemiological data, including clinical and genetic characteristics, longitudinal trends in estimated glomerular filtration rate (eGFR) and urine protein, and the impact of renin-angiotensin system (RAS) inhibitors on the clinical course, using baseline data from our registry.

## Materials and methods

### Study population and data sources

The Japanese ALPort Syndrome (JP-ALPS) registry was established in 2022 to collect information regarding the clinical characteristics, genetics, and treatment of Alport syndrome; it was also conducted to examine the clinical course, long-term prognosis, and genotype–phenotype correlations among affected individuals. Patients with persistent hematuria whose diagnosis was confirmed by genetic or histopathological examination, or whose relatives had a confirmed diagnosis of Alport syndrome were included in the registry. Patients were excluded if they had heterozygous mutations in the *COL4A3* or *COL4A4* gene with hematuria without proteinuria, normal kidney function, and a family history of only hematuria. As of April 2024, 51 pediatric nephrology centers in Japan participated in this registry, and 148 patients were included. The JP-ALPS database includes variables such as age, sex, family history, histopathological findings, gene mutations, laboratory data, medications used, ocular complications, hearing data, esophageal leiomyoma, and outcomes (e.g., kidney replacement therapy).

Among the 148 patients in the registry, we excluded 24 who had missing information regarding whether a biopsy or genetic test had been performed, as well as three with presumably erroneous genetic test results. The final analytical cohort for this study consisted of 121 patients.

The registry enrolls patients of all ages. In addition, blood and urine test results from the onset are collected retrospectively and prospectively every year. In the present study, baseline data were collected at enrolment, and only retrospective data were collected and analyzed.

### Statistical analysis

Patient characteristics were expressed as number (%) or median and interquartile range (IQR). eGFR values were calculated using the age-appropriate eGFR formulas based on serum creatinine (Cr) levels for Japanese children [15, 16]. A spaghetti plot was created to visualize the change in eGFR and urine protein-to-creatinine ratio over time according to age for each patient. Patients with eGFR or urine protein-to-creatinine measurements were included in these analyses. Longitudinal trends for eGFR and urine protein-to-creatinine ratio were estimated using a restricted cubic spline curve with three knots located at the 10th, 50th, and 90th percentiles for age. The slope of eGFR over time before and after RAS inhibitor treatment was estimated using a two-piecewise random coefficient model with a node at the start of RAS inhibitor treatment. For these analyses, patients with available eGFR or urine protein-to-creatinine data both before and after RAS inhibitor administration were included.

Analyses were performed using SAS Version 9.4 (SAS Institute Inc., Cary, NC, USA.) and R version 4.3.1 (www.r-project.org).

## Results

### Patient characteristics

Of the 121 patients included, 105 (86.8%) underwent genetic testing and 82 (67.8%) had a kidney biopsy. All patients received a diagnosis based on either genetic mutations or pathological findings. Patient characteristics are summarized in Table 1. Fifty-eight patients (47.9%) were male; the median ages at disease onset and diagnosis were 3.0 years (IQR 1.0–4.0) and 5.1 years (IQR 3.4–10.8), respectively. Eighty-five patients (70.2%) had a family history of nephritis or chronic kidney disease. Among the 105 patients who underwent genetic testing, 77 (73.3%) had XLAS (39 male and 38 female), eight (7.6%) had ARAS, and 13 (12.4%) had ADAS. Seven cases were missing or had unknown genetic information. The age at detection was lower among patients with XLAS. The age at diagnosis was lowest in male patients with XLAS and highest in patients with ADAS. The median creatinine-based eGFR and urine protein-to-creatinine ratio at onset were 112.9 (IQR 99.3–131.1) mL/min/1.73 m^2^ and 0.35 (IQR 0.19–0.59) g/g Cr, respectively. Kidney function was normal in both male and female patients with XLAS. Urine protein levels were slightly higher in male patients with XLAS than in female patients with XLAS. Eighty-one patients (66.9%) used RAS inhibitors. Among them, 41 patients used angiotensin-converting enzyme inhibitors and 57 patients used angiotensin II receptor blockers. Male patients with XLAS or ARAS were more likely to receive RAS inhibitor treatment. Five patients used sodium-glucose cotransporter 2 inhibitors and one patient used bardoxolone methyl. Six patients transitioned to end-stage kidney disease (ESKD) during the follow-up period.

**Table 1:** Patient characteristics according to genotype

	All	XLAS female	XLAS male	ADAS	ARAS	Unknown
*N* (%)	121	38 (31.4)	39 (32.2)	13 (10.4)	8 (6.6)	23 (19.0)
Age at onset (years)	3.0 [1.0–4.0]	2.5 [1.0–3.0]	2.0 [1.0–3.0]	5.0 [3.0–14.0]	4.5 [2.5–6.2]	3.0 [2.0–3.0]
Age at diagnosis (years)	5.1 [3.4–10.8]	6.6 [4.0–10.3]	3.7 [3.0–8.4]	17.8 [8.1–44.2]	10.1 [5.2–21.0]	4.3 [3.7–7.1]
Male (%)	58 (47.9)	0 (0.0)	39 (100.0)	7 (53.8)	1 (12.5)	11 (47.8)
Receiving kidney biopsy (%)	82 (67.8)	17 (44.7)	26 (66.7)	10 (76.9)	8 (100.0)	21 (91.3)
Receiving gene test (%)	105 (86.8)	38 (100.0)	39 (100.0)	13 (100.0)	8 (100.0)	7 (30.4)
Family history of nephritis/CKD (%)	85 (70.2)	26 (68.4)	31 (79.5)	10 (76.9)	4 (50.0)	14 (60.9)
Use of RAS inhibitors (%)	81 (66.9)	25 (65.8)	31 (79.5)	7 (53.8)	5 (62.5)	13 (56.5)
eGFR at onset (mL/min/1.73m^2^)	112.9 [99.3–131.1]	111.9 [89.4, 122.7]	110.5 [99.7, 132.5]	116.1 [105.3, 122.6]	131.5	121.2 [116.7, 125.8]
Urine protein-to-creatinine ratio at onset (g/gCr)	0.35 [0.19, 0.59]	0.27 [0.17, 0.47]	0.31 [0.22, 0.62]	0.32 [0.20, 4.90]	0.47	5.04 [2.85, 7.22]
ESKD during follow-up period	6 (5.0)	0 (0.0)	1 (2.6)	0 (0.0)	1 (12.5)	4 (17.4)

*XLAS* X-linked Alport syndrome, *ADAS* autosomal dominant Alport syndrome, *ARAS* autosomal recessive Alport syndrome, *CKD* chronic kidney disease, *RAS* renin-angiotensin system, *eGFR* estimated glomerular filtration rate, *ESKD* end-stage kidney disease

### Longitudinal trends in eGFR

**Fig. 1:**
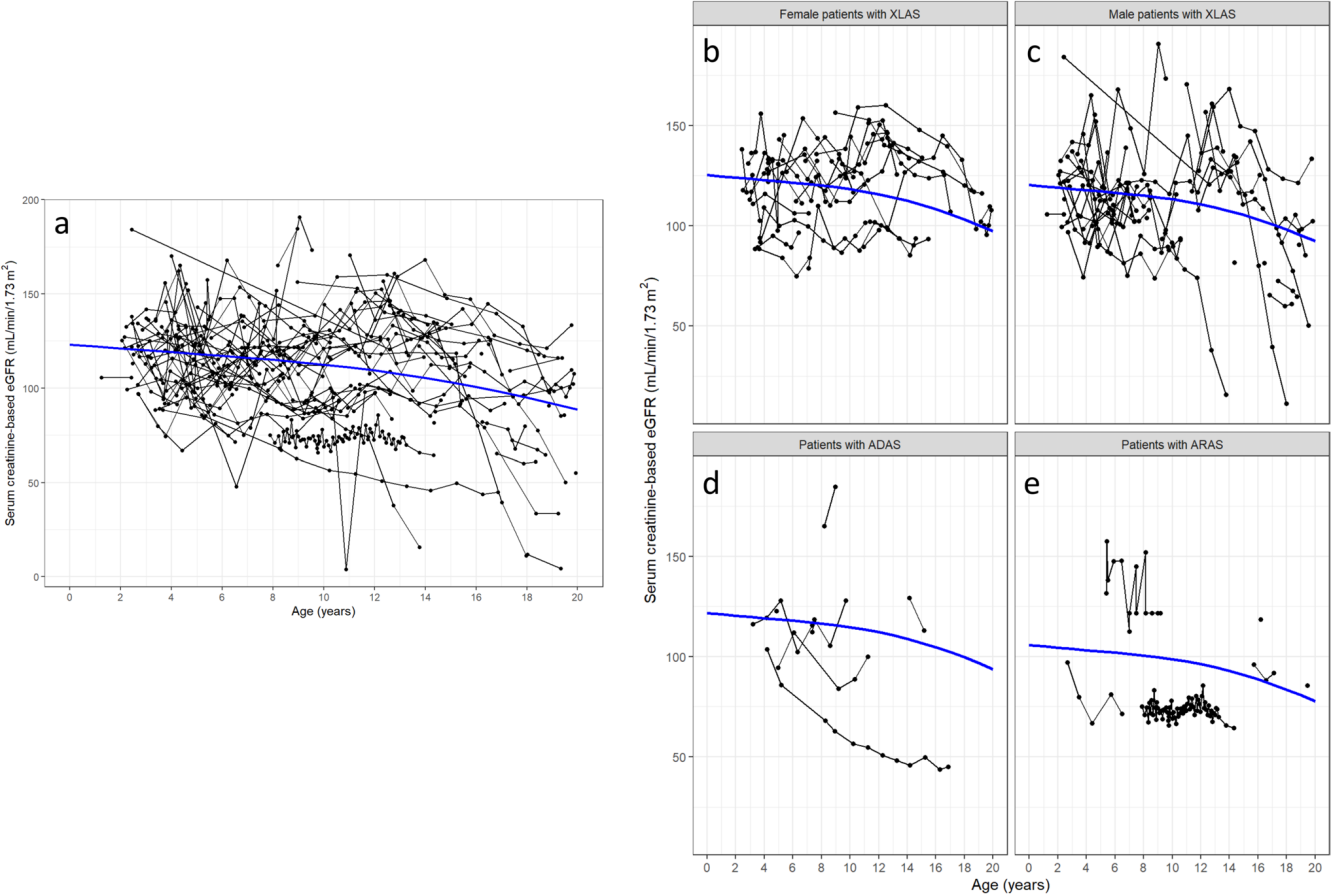
Age-related changes in creatinine-based eGFR among **a** all patients (*n* = 108), **b** female patients with XLAS (*n* = 32), **c** male patients with XLAS (*n* = 35), **d** patients with ADAS (*n* = 12), and **e** patients with ARAS (*n* = 8). Black lines: eGFR values for individual patients. Blue line: restricted cubic spline curve with three knots located at the 10th, 50th, and 90th percentiles for age

Among 108 patients with available eGFR data, eGFR declined slowly with increasing age; the point estimate of eGFR at age 20 was 88.6 mL/min/1.73 m^2^ (Fig. 1a). When stratified according to genotype, eGFR levels and their rates of decline were similar across groups, except for ARAS; however, eGFR levels were slightly better in female patients with XLAS than in patients with other genotypes (Fig. 1b–e). A steep decline in eGFR during adolescence was observed in some male patients with XLAS (Fig. 1c). The same trend was observed when eGFR was calculated using serum cystatin C levels among 87 patients with available cystatin C data (Supplementary Figure).

**Fig. 2:**
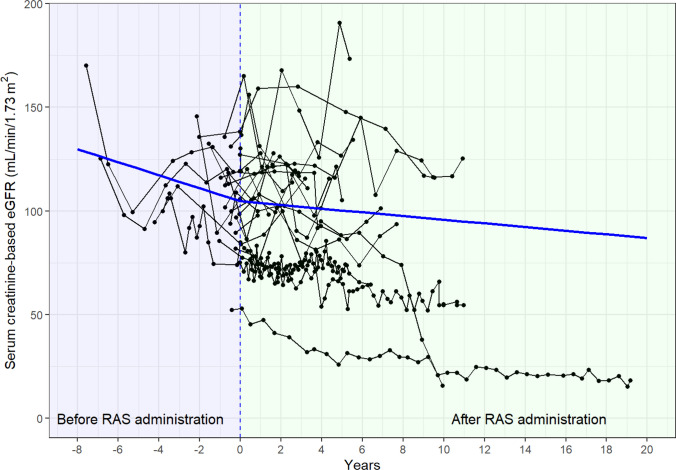
Temporal changes in serum creatinine-based eGFR, before and after initiation of RAS inhibitor treatment (*n* = 23). Black lines: eGFR values for individual patients. Blue line: estimated regression from a two-piecewise random coefficient model, with a node at the start of RAS inhibitor treatment

Among 23 patients who used RAS inhibitors and had available eGFR data before and after RAS inhibitor administration, the rate of eGFR decline was slower after RAS inhibitor initiation (Fig. 2).

### Longitudinal analysis of urine protein

**Fig. 3:**
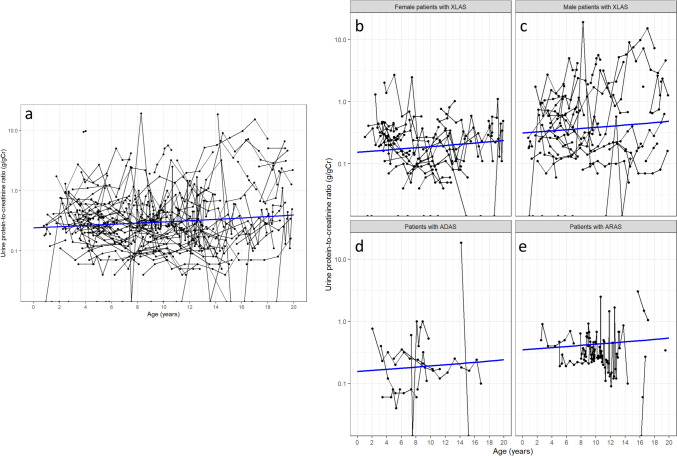
Age-related changes in urine protein-to-creatinine ratio among **a** all patients (*n* = 114), **b** female patients with XLAS (*n* = 36), **c** male patients with XLAS (*n* = 37), **d** patients with ADAS (*n* = 12), and **e** patients with ARAS (*n* = 8). Black lines: urine protein-to-creatinine ratio values for individual patients. Blue line: restricted cubic spline curve with three knots located at the 10th, 50th, and 90th percentiles for age

Among 114 patients with available urine protein-to-creatinine data, the urine protein-to-creatinine ratio increased slowly with advancing age (Fig. 3a). When stratified according to genotype, urine protein levels were slightly higher in male patients with XLAS and all patients with ARAS than in female patients with XLAS and all patients with ADAS (Fig. 3b–e). Massive proteinuria was observed in some male patients with XLAS (Fig. 3c).

**Fig. 4:**
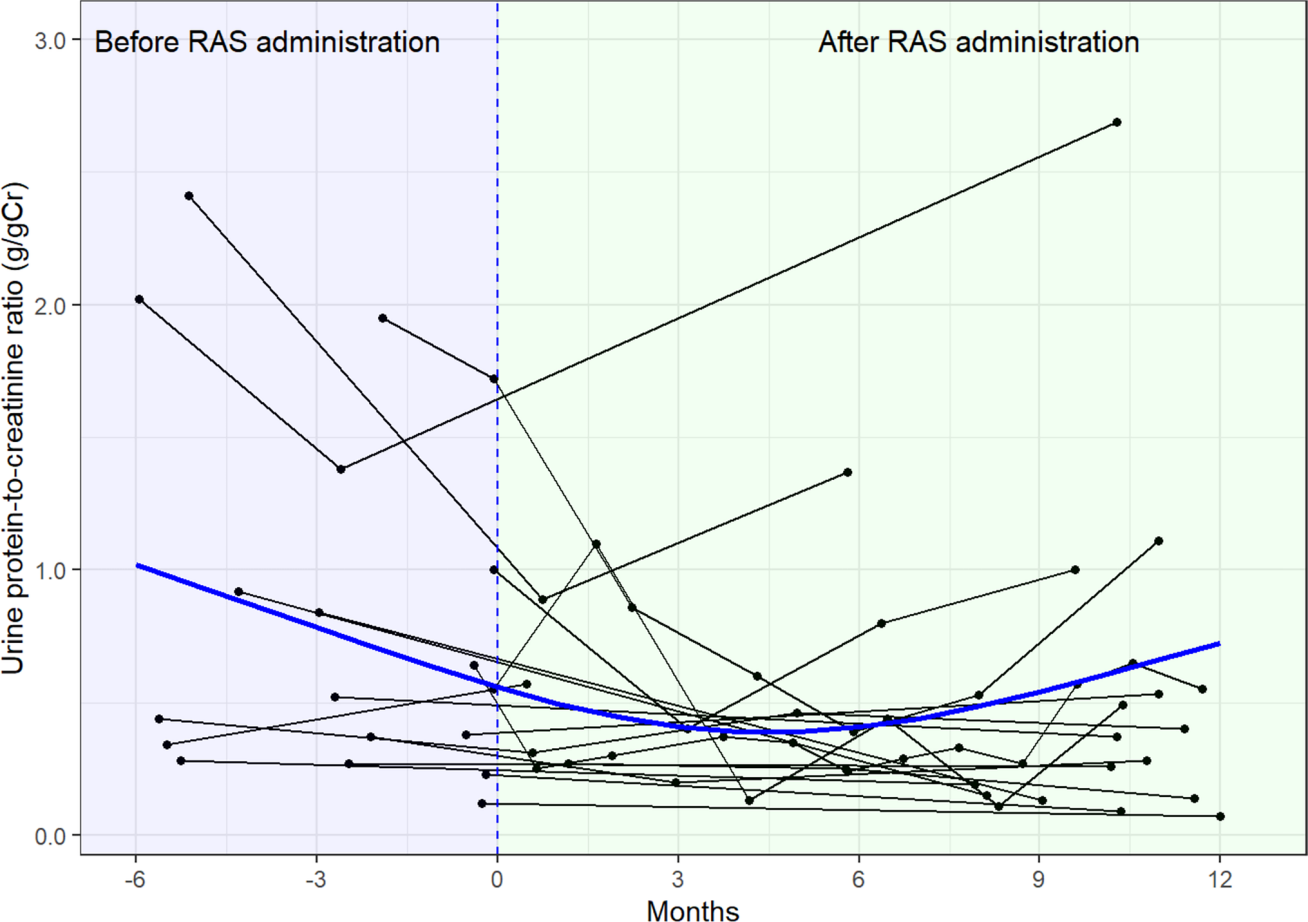
Temporal changes in urine protein-to-creatinine ratio within 1 year after initiation of RAS inhibitor treatment (*n* = 18). Blue line: restricted cubic spline curve with three knots located at the 10th, 50th, and 90th percentiles for age

Although there was a temporary decrease, urine protein levels increased within 1 year after RAS inhibitor administration among 18 patients with available urine protein-to-creatinine ratio data before and after RAS inhibitor administration (Fig. 4).

## Discussion

In this national Alport syndrome cohort in Japan, most patients received a diagnosis through genetic testing. Among those tested, 70% had XLAS. Kidney function was normal at disease onset; eGFR decline was relatively slow during childhood and adolescence. Most patients used RAS inhibitors, and a tendency toward Suppression of eGFR decline by RAS inhibitors was Suggested. Urine protein levels temporarily decreased after RAS inhibitor administration but increased again within 1 year.

As anticipated, XLAS was the most common type of Alport syndrome. There was almost no difference in the number of male and female patients. The proportion of patients with ARAS was lower, whereas the proportion of patients with ADAS was higher, than previously reported (ARAS 15%, ADAS 5%) [2]. The increase in the proportion of patients with ADAS is likely due to improved recognition of this disease, which is characterized by slow progression and no specific pathological findings other than a thin basement membrane. Additionally, advances in genetic diagnosis have strongly contributed to the increased identification of ADAS [17].

Kidney function was normal in both male and female patients with XLAS at disease onset, and the decline in eGFR was mild until age 20 in both groups, though a steep decline in eGFR during adolescence was observed in some male patients with XLAS. Although male patients with XLAS typically progress to ESKD earlier than female patients with XLAS [18, 19], both male and female patients with XLAS in our study appeared to have relatively preserved kidney function throughout childhood and adolescence, potentially due to RAS inhibitor treatment. Jais et al. reported that 51% of male patients develop ESKD by age 20 [20]. At the time of that study, RAS inhibitor treatment was rarely used for Alport syndrome patients. In our cohort, most patients with hematuria and proteinuria had already started RAS inhibitor treatment. This finding indirectly suggests that the treatment successfully slows progression to ESKD. In the present study, we observed a tendency toward a slower rate of eGFR decline following the initiation of RAS inhibitor treatment. This observation is consistent with prior reports and may suggest a potential benefit of early intervention. Although our findings are based on limited analysis in retrospective baseline data from the JP-ALPS registry, we strongly anticipate that similar results will be confirmed in future prospective studies.

In contrast to the similarity in eGFR, proteinuria was more pronounced in male patients with XLAS than in female patients with XLAS. Notably, some male patients with XLAS experienced massive proteinuria. Because proteinuria is a potential risk factor for ESKD in Alport syndrome [21], proteinuria and eGFR levels must be continuously monitored in registered patients to elucidate the association between urine protein levels and kidney function. Urine protein levels also temporarily decreased with RAS inhibitor treatment, but increased again within 1 year. Although this trend may appear contradictory to the observed slowing of eGFR decline, previous studies have shown that partial or transient reductions in proteinuria may still confer long-term renoprotective effects in patients with Alport syndrome [22]. Even in a randomized controlled trial, eGFR decline was attenuated despite modest or variable effects on proteinuria [23].

The ages at disease onset and diagnosis in our cohort were younger than the ages reported in other countries. For example, a nationwide study in Finland showed that the median age at onset was approximately 6 years old [24]. Earlier disease detection in our cohort is undoubtedly attributable to the widespread urinalysis screening program in Japan, which begins at age 3. Because early initiation of RAS inhibitors is recommended to delay the need for kidney replacement therapy, early detection and diagnosis of Alport syndrome are essential [25]. Indeed, most of our patients had a prescription for an RAS inhibitor, and the rate of eGFR decline was slower after RAS inhibitor initiation. Our registry is expected to provide further indirect evidence Suggesting that the widespread urinalysis screening program, initiated at age 3, leads to early detection of Alport syndrome and is effective for improving the kidney prognosis of patients with this disease.

Some important limitations should be acknowledged. First, the participating facilities in the JP-ALPS registry consist primarily of pediatric nephrology centers. Therefore, patients with ESKD at an early age may not be enrolled in the current retrospective study since they may have transitioned to dialysis clinics or transplant centers, resulting in a potential bias that the eGFR trajectories presented here may be better than those obtained from the prospective cohort. In addition, the absence of a comparison group of untreated patients and the retrospective nature of our analysis limit our ability to draw causal inferences regarding the effect of RAS inhibitor treatment. Nevertheless, our results, including eGFR trajectories, would be reasonable among those who did not require kidney replacement therapy in childhood and adolescence. Given that most of the patients develop ESKD in adulthood even in male patients with XLAS [19], we believe that the results presented here are applicable and useful to many patients. Nevertheless, we are continuing to prospectively enroll patients with Alport syndrome and will provide more accurate clinical courses for patients with Alport syndrome in the future in a prospective cohort. Additional data collected in the registry, such as histopathological findings, ocular complications, and hearing data, will be published in future reports as well. Second, because Japanese health insurance does not cover frequent cystatin C measurements, we presented eGFR data primarily based on serum creatinine. Cystatin C-based eGFR is included as a preliminary result in the supplementary material. In addition, the number of time-series data points for eGFR and urine protein-to-creatinine ratio was limited in certain subgroups, which may have led to underfitting in the restricted cubic spline curves. Therefore, these curves should be interpreted with caution. Third, the decision to perform genetic testing depends on individual physicians. This may have influenced the proportions of genotypes observed, particularly regarding ADAS.

In conclusion, we have established a national registry database for Alport syndrome in Japan and presented baseline clinical characteristics of included individuals. Most patients were diagnosed through genetic testing, and the proportion of patients with ADAS was higher than previously reported. In our cohort, which included patients identified early through Japan’s urinalysis screening system and who did not require kidney replacement therapy in childhood or adolescence, kidney function was preserved throughout this period. RAS inhibitor use may be associated with a reduced rate of eGFR decline.

## Supplementary Information

Below is the link to the electronic supplementary material.

Supplementary file1 (PDF 243 KB)
