# Supplementary material for: Investigation of clinical and genetic characteristics of Alport syndrome using a national registry in Japan (JP-ALPS)
Source: Clin Exp Nephrol. 2025 Sep 19;30(1):87–95. doi: 10.1007/s10157-025-02758-w (PMC12811307; doi:10.1007/s10157-025-02758-w)

Supplemental Figure Age-related changes in cystatin-based eGFR among (a) all patients (n = 87), (b) female patients with XLAS (n = 30), (c) male patients with XLAS (n = 30), (d) patients with ADAS (n = 10), and (e) patients with ARAS (n = 6). Black line: eGFR values per patient; Blue line: restricted cubic spline curve with 3 knots located at the 10th, 50th, and 90th percentile of age.

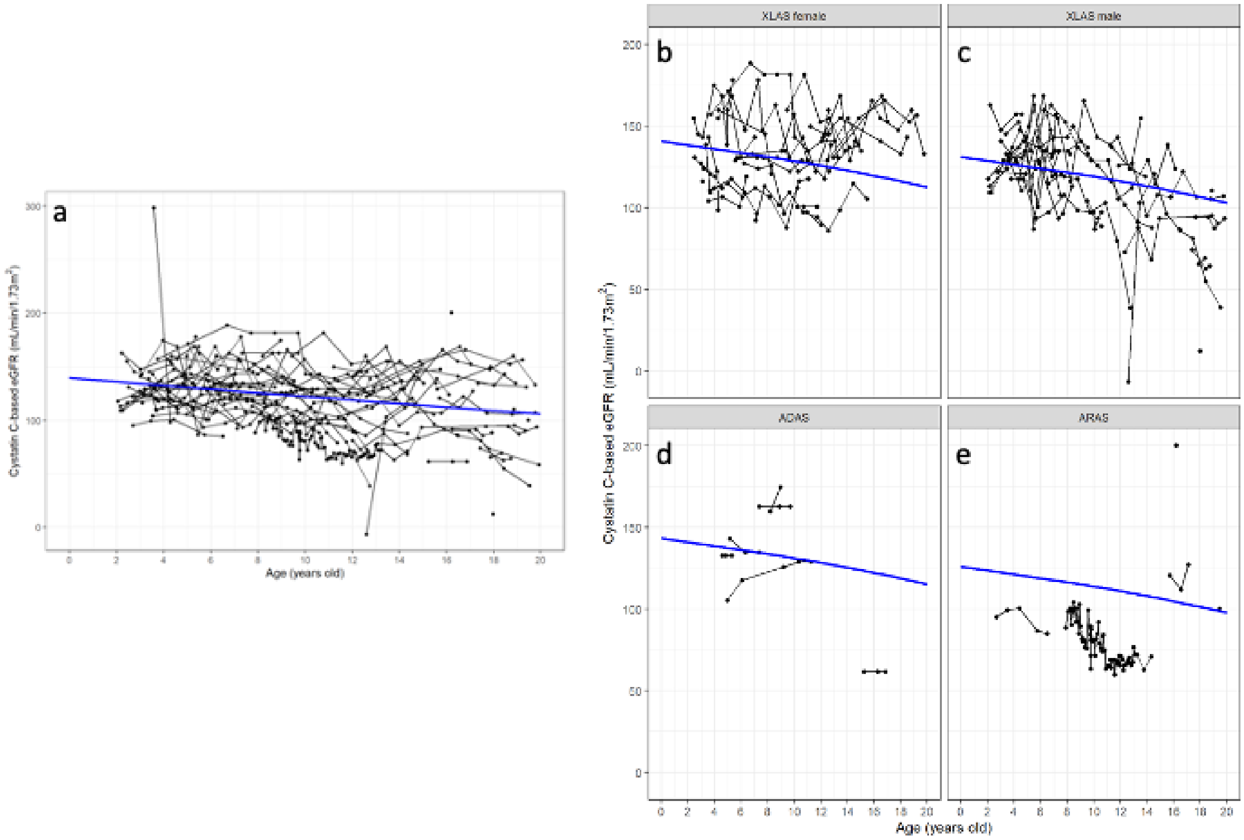

Supplement: Supplementary file 1 — Supplementary file1 (PDF 243 KB) [file 10157_2025_2758_MOESM1_ESM.pdf]
